# Editorial: Challenges of COVID-19 in dermatology patients on immunosuppression: risk, outcome, vaccination and beyond, volume II

**DOI:** 10.3389/fmed.2023.1268285

**Published:** 2023-08-24

**Authors:** Aikaterini Patsatsi, Maryam Daneshpazhooh, Soheil Tavakolpour, Hamidreza Mahmoudi, Dedee F. Murrell

**Affiliations:** ^1^Autoimmune Skin and Bullous Diseases Unit, 2nd Dermatology Department, Aristotle University School of Medicine, Papageorgiou General Hospital, Thessaloniki, Greece; ^2^Department of Dermatology, Autoimmune Bullous Diseases Research Center, Tehran University of Medical Sciences, Tehran, Iran; ^3^Dana-Farber Cancer Institute, Harvard Medical School, Boston, MA, United States; ^4^Department of Dermatology, St George Hospital, University of New South Wales, Sydney, NSW, Australia

**Keywords:** COVID-19, SARS-CoV-2, psoriasis, vaccination, autoimmune bullous diseases, vitamin D, rituximab, pregnancy

In volume II of the Research Topic on “*Challenges of COVID-19 in dermatology patients on immunosuppression: risk, outcome, vaccination and beyond - volume II*” the impact of COVID−19 vaccination in patients with autoimmune skin diseases is further discussed.

In the literature, there are several articles describing the first presentation or the exacerbation of autoimmune skin diseases after vaccination against COVID-19.

Karampinis et al. performed a retrospective study in psoriasis patients after vaccination to detect disease flare ups and to evaluate whether insufficiency of vitamin D may facilitate this specific scenario. They concluded that patients with insufficient levels of vitamin D are more prone to postvaccination aggravation of psoriasis.

Similar considerations about the aggravation of autoimmune bullous diseases after COVID 19 vaccination are discussed in the paper by Kianfar et al.. In this study the vaccine safety was evaluated after contact with the patients to determine their status before and after vaccination.

According to the authors, for every three patients who received vaccines during the active phase of the disease one experienced disease exacerbation. Although there is a risk of exacerbation of the blistering disease after vaccination, it is not that high to prevent patients from getting the vaccine. As a rule, it seems that the risk of relapse is even lower when the patient is in remission and well controlled before vaccination.

Rituximab is one of the drugs that raised concerns during the pandemic. Physicians were reluctant to treat pemphigus patients with rituximab due to its long-term B-cell suppression. Additionally, there were many considerations regarding the time and efficacy of vaccination. Koszegi et al. present three cases of pemphigus patients who had been treated with rituximab and received COVID-19 vaccinations prior to becoming infected with COVID-19. The course of infection was mild, underlining the importance of full vaccination in patients with pemphigus prior to rituximab treatment.

Treatment of autoimmune diseases with immunomodulatory drugs or biologics in pregnant women with autoimmune skin diseases was also challenging during the pandemic. Messas et al. reviewed the evidence regarding continuing immunomodulatory therapy in pregnant patients during the pandemic and found that there is no compelling reason for treating them differently than non-pregnant women. Based on data from pregnant patients with rheumatologic diseases, vaccination against COVID-19 seems not only safe, but it may improve the obstetric outcome. Thus, COVID-19 vaccination is recommended in pregnant patients under immunomodulatory agents.

Overall, physicians treating patients with autoimmune skin diseases should encourage them to follow their recommended treatment and receive the COVID 19 vaccines whenever needed.

## Author contributions

AP: Conceptualization, Data curation, Supervision, Writing—original draft, Writing—review and editing. MD: Conceptualization, Data curation, Supervision, Writing—original draft, Writing—review and editing. ST: Conceptualization, Writing—review and editing. HM: Conceptualization, Data curation, Writing—review and editing. DM: Conceptualization, Data curation, Supervision, Writing—original draft, Writing—review and editing.

## Dedication

This issue is dedicated to the challenges dermatology patients have been facing during the COVID-19 pandemic.

